# Arginase-1-expressing macrophages are dispensable for resistance to infection with the gastrointestinal helminth *Trichuris muris*

**DOI:** 10.1111/j.1365-3024.2011.01300.x

**Published:** 2011-07

**Authors:** R Bowcutt, L V Bell, M Little, J Wilson, C Booth, P J Murray, K J Else, S M Cruickshank

**Affiliations:** 1 Faculty of Life Sciences, University of ManchesterManchester, UK; 2 Epistem LimitedManchester, UK; 3 Departments of Infectious Diseases and Immunology, St. Jude Children’s Research HospitalMemphis, TN, USA

**Keywords:** arginase, helminth, macrophage

## Abstract

Alternatively activated macrophages (AAMs) have key roles in the immune response to a variety of gastrointestinal helminths such as *Heligmosomoides bakeri* and *Nippostrongylus brasiliensis*. In addition, AAMs have been implicated in the resolution of infection-induced pathology in *Schistosoma mansoni* infection. AAMs exert their activity in part via the enzyme arginase-1 (Arg1), which hydrolyses l-arginine into urea and ornithine, and can supply precursor substrate for proline and polyamine production. *Trichuris muris* is a worm that resides in the large intestine with resistance being characterized by a Th2 T-cell response, which drives alternatively activated macrophage production in the local environment of the infection. To investigate the role of AAMs in *T. muris* infection, we used independent genetic and pharmacologic models of arginase deficiency. In acute infection and Th2-dominated immunity, arginase-deficient models expelled worms normally. Macrophage-Arg1-deficient mice showed cytokine and antibody levels comparable to wild-type animals in acute and chronic infection. We also found no role for AAMs and Arg1 in infection-induced pathology in the response to *T. muris* in either chronic (Th1 dominated) or acute (Th2 dominated) infections. Our data demonstrate that, unlike other gastrointestinal helminths, Arg1 expression in AAMs is not essential for resistance to *T. muris* in effective resolution of helminth-induced inflammation.

## Introduction

Macrophages play pivotal roles in both innate and adaptive immune responses through phagocytosis, antigen presentation and direct pathogen killing. In addition to host protection, macrophages are also involved in the maintenance of gut homeostasis, the resolution of pathology and tissue repair (1). The cytokine milieu is thought to lead to the development of two broad subsets of macrophage (2). Classically activated macrophages (CAMs) have been shown to ‘develop’ in a Th1 environment with IFN-γ playing a significant activation role (2). In addition, TNFα and microbial products such as lipopolysaccharide influence classical activation (3). CAMs exert their protective role against intracellular pathogens through l-arginine metabolism and the subsequent production of nitric oxide (3). In contrast to CAMs, STAT6-dependent alternative activation occurs in the presence of IL-4 or IL-13, produced by Th2 cells and innate immune cells such as mast cells (3). Alternatively activated macrophages (AAMs) are characterized by the up-regulation of the cell surface receptors IL4Rα chain and mannose receptor, the expression of the genes *Arg1, Retnla* (encoding Fizz1/RELMα) and *Chi3l3* (Ym1), and the transcription factor PPARγ (3).

Along with their role in tissue repair, and because of their development in a Th2-rich environment, AAMs have been hypothesized to play an important role in immunity to extracellular pathogens such as helminths (4). Immunity to helminths is mediated by CD4^+^ T cells, with a Th1 response associated with susceptibility to infection and a Th2 response associated with parasite expulsion and resistance (5). Previous research has shown AAMs to be present in most helminth infections. The numbers of circulating AAMs increase in mice upon infection with the small-intestinal parasite *Nippostrongylus brasiliensis*, and the infection-associated alterations of the gastrointestinal smooth muscle are thought to be AAMØ dependent (6). *Nippostrongylus brasiliensis* expulsion has also been shown to be impaired after clodronate-mediated depletion of macrophages or after blocking arginase activity by pharmacologic agents (6). Alternatively activated macrophages are thought to be important in infections with other nematodes such as *Brugia malayi* and *Litomosiodes sigmodontis*, where infection induces the recruitment of F4/80^+^ cells, along with the up-regulation of the associated alternatively activated genes, *Retnla* (RELMα/FIZZ1) and *Chi3l3* (Ym1), at the site of infection (7). Furthermore, depletion of macrophages or blocking arginase activity with the inhibitor (S)-(2-Boronethyl)-L-cysteine (BEC) in mice infected with *Heligmosomoides bakeri* (formerly *Heligmosomoides polygyrus)* results in increased parasite burdens (8). Previous research has therefore suggested a role for AAMs in a range of parasitic infections as possible effector cells.

We aimed to define the role of the AAMs in resistance to the large-intestinal parasite *Trichuris muris*. Resistance to *T. muris* infection is associated with a dominant Th2 response characterized by IL4, IL13, IL9, IL5 and susceptibility, a Th1 response characterized by IFN-γ and IL-12 (5). Alternatively activated macrophages have been shown to be present in the caecum and proximal colon of *T. muris*-infected, resistant C57BL/6 mice around the time of parasite expulsion. We used the mice lacking Arg1 in macrophages (9), where the floxed arginase-1 (*Arg1*) gene is deleted in all haematopoietic and endothelial cell lineages. Arg1 is expressed in myeloid and not lymphoid lineages; therefore, *Arg1*^flox/flox^;*Tie2*-cre mice are used as a model of Arg1 deficiency in macrophages (9). In addition, we used C57BL/6 mice treated with the arginase inhibitor L-2-Amino-(4-(2′hydroxyguanidino) butyric acid (nor-NOHA) (10), which inhibits the activity of both arginase 1 and arginase 2. We measured parasite expulsion kinetics along with several parameters of gut pathology in both these mouse models. Our data therefore suggest that arginases are not essential for resistance to *T. muris* and, in addition, are not crucial for the effective resolution of helminth-induced pathology.

## Materials and methods

### Mice

Male *Arg1*^*flox/flox*^*;Tie2-cre* and control *Arg1*^*+/+*^*;Tie2-cre e* (11) mice have been described and were bred in-house (9,12). All mice were routinely screened by PCR to confirm their genotype *Arg1*^*flox/flox*^*;Tie2-cre* (9). PCR was performed on ear punches using TaqGold and buffers (Applied Biosystems, Paisley, UK). Primer sequences were as follows: floxed allele; 5′-TGCGAGTTCATGACTAAGGTT-3′ 5′-AAAGCTCAGGTGAATCGG-3′, Tie2cre; 5′-CGCATAACCAGTGAA ACAGCATTGC-3′ 5′ CCCTGTGCTCAGACAGAAATGA G A-3′, Delta allele; 5′-CCCCCAAAGGAAATGTAAGAA-3′ 5′-CACTGTCTAAG CCCGA G AGTA-3′. Specific pathogen-free male C57BL/6 mice were purchased at 6–8 weeks of age from Harlan Olac (Bicester, UK). All mice were maintained by the Biological Services Unit, University of Manchester, UK, and kept in individually ventilated cages. Animals were treated and experiments performed according to the Home Office Animals (Scientific Procedures) Act (1986).

### Parasites

Maintenance of the *T. muris* life cycle and production of excretory/secretory (E/S) antigen was carried out as described previously (13). Mice were infected with approximately 175 embryonated eggs by oral gavage and killed at various timepoints post-infection (p.i.), when worm burdens were assessed as described previously (14,15).

### Parasite-specific antibody ELISA

*Trichuris muris*-specific IgG1 and IgG2a were measured in serum samples collected at autopsy by ELISA using a previously described method (16).

### Histology

Caecal snips were fixed in neutral-buffered formalin for 24 h, processed and embedded in paraffin wax. Five micrometre sections were then dewaxed, rehydrated and stained using a standard haematoxylin & eosin, periodic acid Schiff or Gomori’s one-step trichrome stain method. Crypt length was measured in 20 crypts per mouse from H&E-stained sections using WCIF imagej software (available from http://rsbweb.nih.gov/ij/index.html). Goblet cells were counted in 20 crypts per mouse from periodic acid-Schiff stained (PAS-stained) sections. All slides were measured and counted in a blind, randomized order.

Expression of arginase and RELMα was assessed in gut caecum tissue by immunohistochemistry. Slides of paraffin-embedded tissue were dewaxed and rehydrated. Endogenous peroxidases were quenched by incubation for 20 min in 30% H_2_O_2_ in methanol for anti-arginase-stained samples and 1.5 μL/mL glucose oxidase (Sigma Aldrich, Dorset, UK) for Relmα-stained sections. Antigen retrieval was performed using pepsin digest solution (Invitrogen, Paisley, UK). Sections were blocked with rat serum for 1 h, and endogenous biotins were blocked using the avidin/biotin blocking kit as per the manufacturers’ instructions (Vector Laboratories Ltd, Peterborough, UK). For arginase1 staining, only slides were incubated with the mouse on mouse (M.O.M) Ig-blocking reagent for 1 h. Stock M.O.M diluent was added to the slides for 5 min. Sections were incubated with primary antibodies to arginase1 (Becton Dickinson, Oxford, UK) diluted in M.O.M diluent or anti-RELMα (R and D Systems, Abingdon, UK) diluted in PBS. For RELMα staining, only a secondary antibody-biotinylated goat anti-rat IgGF(ab)_2_ (Chemicon International, Watford, UK) was used. Slides were incubated with avidin and biotinylated horseradish peroxidase macromolecular complex kit (ABC; Vector laboratories), for 30 min. 3, 3′Diaminobenzidine (substrate for peroxidase, Vector Laboratories) was added to samples and the colour development monitored under a microscope. Slides were washed and counterstained with HaemQS, washed and mounted. The number of arginase positive RELMα-positive cells was quantified in a blind randomized order.

### Mesenteric lymph node cell culture

Single cell suspensions were prepared from mesenteric lymph nodes (MLNs) taken at autopsy and added at 5 × 10^6^ cells/well in 1-mL cultures to 48-well plates and stimulated with *T. muris* E/S at 50 μg/mL. Cells were incubated at 37°C, 5% CO_2_, 95% humidity for 48 h, after which time supernatants were harvested and stored at −20°C for later cytokine analysis by cytokine bead array (CBA).

### Cytokine bead array

Levels of IL-4, IL-10, IL-6, IL-9, IL-13, interferon gamma, tumour necrosis factor α, IL-12p70 and MCP1 were determined via cytometric bead array (CBA; Becton Dickinson). Briefly, lyophilized cytokine standards were pooled, reconstituted using assay diluent and serial dilutions from 1 : 2 to 1 : 256 prepared. The Protein Flex Set Capture Bead mix and Protein Flex Set Detection Reagent mix were prepared; all beads were pooled allowing 0.3 μL of each bead per well, and beads were reconstituted in the total volume needed in capture bead or detection reagent diluent; 16.5 μL of capture bead mix and 16.5 μL of standard/sample were added to each well; Plates were shaken for 5 min and incubated for 1 h; 16.5 μL of detection bead mixture was added to each well. Plates were incubated for 1 h. Plates were washed and beads re-suspended. Samples were then analysed using BD FacsAria cytometer and fcap array software (Becton Dickinson).

### Statistics

Where statistics are quoted, two experimental groups were compared using the Mann–Witney *U*-test. Three or more groups were compared using the Kruskal–Wallis test, with Dunn’s multiple comparison post-test. *P*-values <0·05 were considered significant. All statistical analyses were carried out using graphpad prism for windows, version 3.02 (GraphPad Software Inc, La Jolla, CA, USA).

## Results

### Expulsion of *Trichuris muris* from BL6 mice does not require Arg1 expression in macrophages

**Figure 1:**
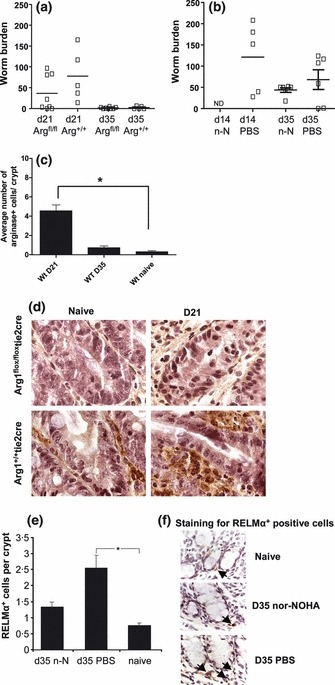
Expulsion of *Trichuris muris* from BL6 mice is not dependent on arginase. Mice were infected orally with approximately 175 embryonated *T. muris* eggs, killed at days 14, 21 and 35 p.i., and worm burden in the caecum and proximal colon assessed. (a) *Arg1*^*flox/flox*^*;Tie 2-cre* (Arg^fl/fl^) and *Arg1*^*+/+*^*;Tie 2-cre* (Arg^+/+^) worm burden at day 21 and 35 p.i. (b) worm burden at day 14 and 35 p.i. nor-NOHA-treated (n-N) and PBS control-treated (PBS) C57BL/6 mice. Data shown are for individual mice (□), with mean values per group (−), and are pooled from two independent experiments, *n* = 5 (*Arg1*^*+/+*^*;Tie 2-cre* mice) and *n* = 8 for (*Arg1*^*flox/flox*^*;Tie 2-cre* mice) (a), and *n* = 5 mice per group (b). ND = not done. Quantification of number of arginase1^+^ cells (arrowed) per crypt in the caecum of naïve, D21 and D35 post-infection in *Arg1*^*+/+*^*;Tie 2-cre* mice (c). No arginase-positive cells were found in *Arg1*^*flox/flox*^*;Tie 2-cre* mice. Representative images of arginase staining are shown in Figure 1d. Quantification of number of RELMα^+^ cells (arrowed) per crypt in naïve, nor-NOHA-treated (n-N) and PBS control-treated (PBS) C57BL/6 mice (e). Representative images of RELMα staining are shown in f.

Male *Arg1*^*flox/flox*^*;Tie2-cre* and control *Arg1*^*+/+*^*;Tie 2-cre* were infected with 175 infective *T. muris* eggs, killed at days 21 and 35 p.i., and worm burdens assessed (Figure 1a). Both *Arg1*^*flox/flox*^*;Tie 2-cre* and *Arg1*^*+/+*^*;Tie 2-cre* mice were able to expel the worms, with almost all mice completely clear of parasites by day 35 p.i. C57BL/6 mice were also infected with around 175 infective *T. muris* eggs and treated with nor-NOHA by i.p. injection up to day 21 post-infection to inhibit all arginases including arginase 1. Mice treated with nor-NOHA expelled their worm burden as efficiently as PBS control-treated animals (Figure 1b). These data suggest that arginase activity in general is not essential for the expulsion of *T. muris*. Arginase-expressing macrophages were rare in the naïve gut but increased significantly around day 21 post-infection (Figure 1c,d). Furthermore, the arginase staining confirmed that there was an absence of arginase in macrophages in the caecum of *Arg1*^*flox/flox*^*;Tie 2-cre* animals (Figure 1c,d). Similar results were observed using RELMα (encoded by *Retnla*), a marker of AAMs, by immunohistochemistry. RELMα-positive cells were increased in the guts of mice post-infection with *T. muris* but reduced in the presence of the arginase inhibitor nor-NOHA (Figure 1e,f).

### *Trichuris muris*-specific cytokine responses are not affected by the absence of arginase

**Figure 2:**
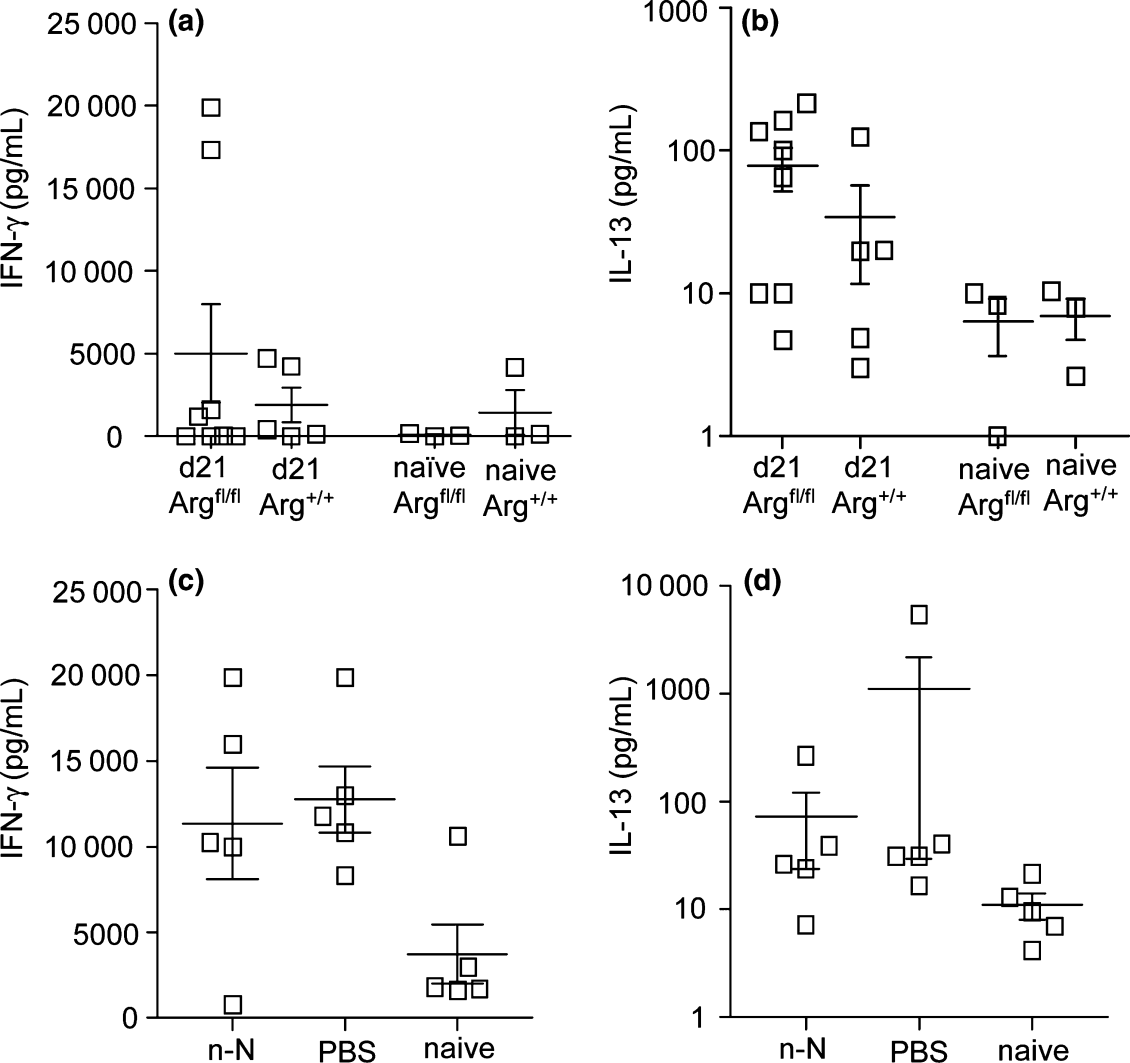
*Trichuris muris*-specific cytokine responses are not affected by the absence of *arginase*. Mice were infected orally with approximately 175 embryonated *T. muris* eggs and mesenteric lymph nodes cells restimulated with *T. muris* E/S at day 21 p.i. IFN-γ and IL-13 levels in supernatants were then assayed by cytokine bead array. *Arg1*^*flox/flox*^*;Tie 2-cre* (Arg^fl/fl^) and *Arg1*^*+/+*^*;Tie 2-cre* (Arg^+/+^) IFN-γ (a) and IL-13 (b) infected versus naïve animals. (c, d) nor-NOHA-treated (n-N) and PBS-treated (PBS) infected C57BL/6 mice versus naïve. Data shown are for individual mice (□), with mean values per group (−), and are pooled from two independent experiments. IFN-γ data are presented on a linear axis, whereas IL-13 data are presented on a log scale. *n* = 3 naïve animals, *n* = 5 (*Arg1*^*+/+*^*;Tie 2-cre*-infected animals), *n* = 8 (*Arg1*^*flox/flox*^*;Tie 2-cre*-infected animals) (a, b) and *n* = 5 mice per group (c, d).

Mesenteric lymph nodes cells from both *Arg1*^*flox/flox*^*;Tie2-cre* and control *Arg1*^*+/+*^*;Tie 2-cre* and nor-NOHA- or PBS-treated C57BL/6 mice at day 21 p.i. were cultured with *T. muris* E/S antigens and the supernatants analysed for IFN-γ and IL-13 by CBA (Figure 2). Levels of IFN-γ were similar between *Arg1*^*flox/flox*^*;Tie2-cre* and *Arg1*^*+/+*^*;Tie 2-cre* controls (Figure 2a) and between nor-NOHA- and PBS-treated BL6 mice (Figure 2c). Also, there were no differences in IL-13 between *Arg1*^*flox/flox*^*;Tie 2-cre* and *Arg1*^*+/+*^*;Tie 2-cre* controls (Figure 2b) or between nor-NOHA- and PBS-treated BL6 mice (Figure 2d). Similarly, no significant differences in MLN-derived IL10 were observed post-infection between control PBS-treated mice (458.3 ± 107.9 pg/mL), and nor-NOHA-treated mice (347.8 ± 267.8 pg/mL) as well as *Arg1*^*+/+*^*;Tie 2-cre*-infected mice (43.4 ± 62.3 pg/mL) and *Arg1*^*flox/flox*^;control *Arg1*^*+/+*^*;Tie 2-cre*-infected mice (196.7 ± 318.3 pg/mL). Furthermore, there were no significant differences in the levels post-infection of CCL2 (MCP1) between control PBS-treated (916.9 ± 554.2 pg/mL) and nor-NOHA-treated groups (780.4 ± 156.4 pg/mL) as well as *Arg1*^*+/+*^*;Tie 2-cre* control mice (355.8 ± 210.3 pg/mL) and. *Arg1*^*flox/flox*^*;Tie2-cre* mice (256.3 ± 239.9 pg/mL). In addition to the cytokines displayed in Figure 2 and those mentioned above, we measured levels of IL-4, IL-6, IL-9, IL-13, interferon γ, tumour necrosis factor α, IL-12p70. No differences were observed between all cytokines analysed; thus, arginases do not seem play an overt role in the regulation of Th-cell responses to *T. muris* infection.

### *Trichuris muris*-specific serum antibody responses are not affected by the absence of arginase activity

**Figure 3:**
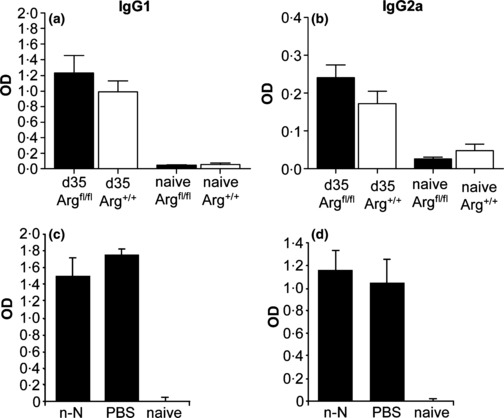
*Trichuris muris*-specific serum antibody responses are not affected by the absence of arginase. Mice were infected orally with approximately 175 embryonated *T. muris* eggs and serum harvested at day 35 p.i. *T. muris*-specific IgG1 and IgG2a levels were measured by ELISA from sera samples diluted at 1 in 80 with PBS (a, b) and at 1 in 160 with PBS (c, d). (a, b) IgG1 and IgG2a levels in *Arg1*^*flox/flox*^*;Tie 2-cre* (Arg^fl/fl^) and *Tie 2-cre* (Arg^+/+^) mice. (c, d) nor-NOHA (n-N) versus PBS-treated C57BL/6 mice. Data shown are mean +/− SEM and are pooled from two independent experiments. *n* = 3 (*Tie 2-cre* and *Arg1*^*flox/flox*^*;Tie 2-cre* naïve animals), *n* = 5 (*Tie 2-cre* animals) *n* = 8 (*Arg1*^*flox/flox*^*;Tie 2-cre*-infected animals) (a, b) and *n* = 5 mice per group (c, d).

Serum from both *Arg1*^*flox/flox*^*;Tie2-cre* and *Arg1*^*+/+*^*;Tie 2-cre* controls and nor-NOHA- or PBS-treated C57BL/6 mice was harvested at day 35 p.i. and levels of *T. muris*-specific IgG1 and IgG2a measured by ELISA (Figure 3). There were no significant differences in levels of IgG1 or IgG2a between *Arg1*^*flox/flox*^*;Tie 2-cre* and *Arg1*^*+/+*^*;Tie 2-cre* controls (Figure 3a,b) or indeed between C57BL/6 mice treated with nor-NOHA and PBS controls (Figure 3c,d).

### Arginase is not essential for the regulation of pathology during *Trichuris muris* infection

**Figure 4:**
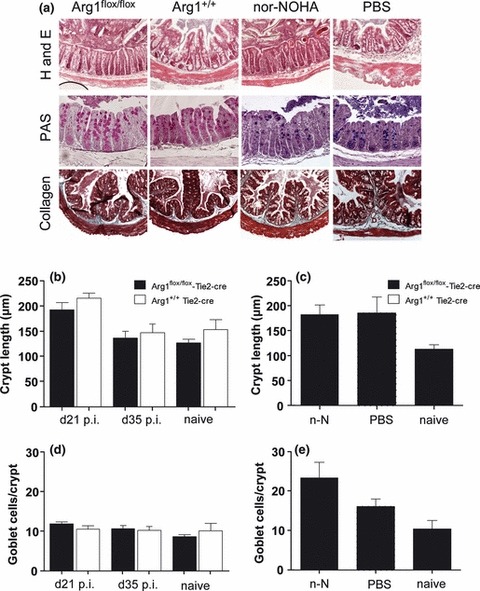
Arginase is not essential for the regulation of pathology during *Trichuris muris* infection. (a) Representative caecal tissue sections stained using H&E, PAS or Gomori’s trichrome of naïve mice and mice infected orally with approximately 175 embryonated *T. muris* eggs. Crypt lengths were assessed using image j software, and 20 crypts per mouse were measured (b, c). b; *Arg1*^*flox/flox*^*;Tie 2-cre* (black bars) and *Arg1*^*+/+*^*;Tie 2-cre* (white bars), c; nor-NOHA-treated (n-N) and PBS-treated (PBS) infected C57BL/6 mice versus naïve. Goblet cells were counted in 20 crypts per mouse, and data are displayed as mean number of goblet cells per crypt (d, e). Data shown are mean + SEM and are pooled from two independent experiments. *n* = 3 (*Arg1*^*+/+*^*;Tie 2-cre* and *Arg1*^*flox/flox*^*;Tie 2-cre* naïve animals), *n* = 5 (*Arg1*^*+/+*^*;Tie 2-cre* animals) *n* = 8 (*Arg1*^*flox/flox*^*;Tie 2-cre*-infected animals) (b, d) and *n* = 5 mice per group (c, e).

We next assessed intestinal inflammation by comparing crypt length and goblet cell numbers from caecum sections taken at autopsy. *Arg1*^*flox/flox*^*;Tie 2-cre* and *Arg1*^*+/+*^*;Tie 2-cre* control mice were sacrificed at days 21 and 35 p.i. and caecal tissue sections stained using H&E (Figure 4a). Crypt lengths were found to be increased in infected mice compared with naïve at day 21 p.i. but there were no obvious differences between *Arg1*^*flox/flox*^*;Tie 2-cre* and *Arg1*^*+/+*^*;Tie 2-cre* controls at either day 21 or 35 p.i. (Figure 4b). Furthermore, goblet cells, stained using PAS, indicated that no overt differences were seen in goblet cell numbers between *Arg1*^*flox/flox*^*;Tie 2-cre* and *Arg1*^*+/+*^*;Tie 2-cre* controls at day 21 or 35 p.i. (Figure 4d). nor-NOHA- and PBS-treated mice were killed at day 35 p.i. and the same pathology parameters measured. Again, crypt length increased post-*T. muris* infection but was equivalent between nor-NOHA and PBS-treated mice (Figure 4c). Similarly, a goblet cell hyperplasia was seen in both infected groups compared with naïve, with no significant differences in goblet cell number between nor-NOHA-treated and PBS-treated infected mice (Figure 4e). In addition, sections were stained for collagen using Gomori’s one-step trichrome stain method. Results show no difference in collagen deposition between *Arg1*^*flox/flox*^*;Tie 2-cre* and *Arg1*^*+/+*^*;Tie 2-cre* controls (Figure 4a). Similar findings were observed in the nor-NOHA-treated and PBS-treated infected mice (Figure 4a). These data suggest that arginase is not critically involved in the regulation of inflammation post-*T. muris* infection.

### Arginase activity is not essential for the regulation of the host immune response and parasite-induced pathology during chronic *Trichuris muris* infection

**Figure 5:**
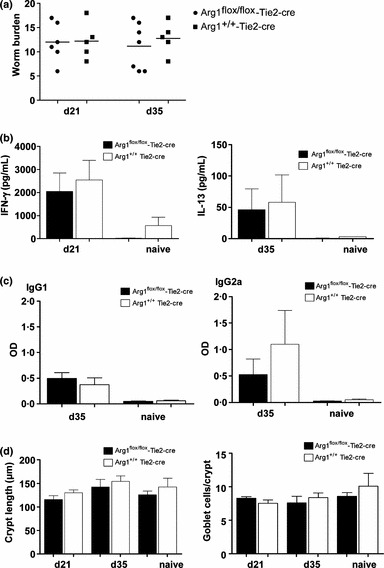
Analysis of the immune response and inflammatory regulation in *Arg1*^*flox/flox*^*;Tie 2-cre* and control mice during chronic *Trichuris muris* infection. *Arg1*^*flox/flox*^*;Tie 2-cre* and *Arg1*^*+/+*^*;Tie 2*-cre mice were infected orally with approximately 20 embryonated *T. muris* eggs and allowed to develop a chronic infection. (a) Worm burdens were assessed in the caecum and proximal colon of *Arg1*^*flox/flox*^*;Tie 2-cre* (circles) and control *Arg1*^*+/+*^*;Tie 2-cre* (squares) at days 21 and 35 p.i. (b) mesenteric lymph nodes cells from naïve controls or infected mice (day 21 p.i) were stimulated with *T. muris* E/S, and levels of IFN-γ and IL-13 in supernatants were analysed by cytokine bead array. (c) Levels of *T. muris*-specific IgG1 and IgG2a were measured by ELISA from sera samples diluted at 1 in 160 with PBS. (d) Representative caecal tissue sections were stained using H&E and PAS. Crypt lengths were analysed (20 crypts per mouse). *Arg1*^*flox/flox*^*;Tie 2-cre* (black bars) and *Arg1*^*+/+*^*;Tie 2-cre* (white bars). Goblet cells were counted in 20 crypts per mouse, and data are displayed as mean number of goblet cells per crypt. Data shown are mean + SEM and are pooled from two independent experiments. *n* = 5 mice for (*Arg1*^*+/+*^*;Tie 2-cre* animals) and *n* = 6-7 (*Arg1*^*flox/flox*^*;Tie 2-cre* mice).

A role for arginase was assessed in chronic *T. muris* infection by administrating mice with a low-dose infection (approximately 20 embryonated *T. muris* eggs). We assessed worm burdens (Figure 5a), cytokine production by restimulated MLNs cells (Figure 5b) and parasite-specific antibody production (Figure 5c). In addition to immunological parameters, we analysed infection-induced pathology by assessing crypt length and goblet cell numbers (Figure 5d). In all parameters assessed, *Arg1*^*flox/flox*^*;Tie 2-cre* mice were equivalent to *Tie 2-cre* controls. Both *Arg1*^*flox/flox*^*;Tie 2-cre* and *Arg1*^*+/+*^*;Tie 2-cre* mice had parasite worm burdens at D35 post-infection consistent with chronic infection. Infected animals showed an increase in IFN-γ production (Figure 5b). In addition, a slight increase in IL-13 levels was observed in both *Arg1*^*flox/flox*^*;Tie 2-cre*- and *Arg1*^*+/+*^*;Tie 2-cre*-infected mice. Levels of parasite-specific IgG1 and IgG2a were low in both *Arg1*^*flox/flox*^*;Tie 2-cre*- and control *Arg1*^*+/+*^*;Tie 2-cre*-infected mice (Figure 5c). Intestinal pathology during chronic infection was assessed by comparing crypt length and goblet cell numbers from caecum sections taken at autopsy. No difference was observed in the average crypt lengths and goblet cell numbers of both *Arg1*^*flox/flox*^*;Tie 2-cre* and *Arg1*^*+/+*^*;Tie 2-cre*-infected mice (Figure 5d). These data suggest that arginase activity in general is not essential for the maintenance of the immune response and the regulation of infection-induced pathology during chronic *T. muris* infection.

## Discussion

Chronic helminth infections such as *H. bakeri* (8) and *N. brasiliensis* (6), typified by a Th2 immune response, are strongly associated with the recruitment of AAMØ, which have been hypothesized to have a role in host resistance. Experimental approaches using clodronate-loaded liposomes to deplete all macrophages or specific inhibitors of arginase function resulted in reduced parasite expulsion in *N. brasiliensis* infection (6) and rendered normally resistant mice susceptible to challenge infection with *H. bakeri* (8). Similarly, resistance to *T. muris* is characterized by a Th2-dominated immune response (5), with BALB/c mice showing Th2-mediated resistance to a high-dose infection and AKR mice showing Th1-mediated susceptibility. We used C57BL/6 mice that, while resistant, characteristically display a mixed Th1/Th2 response to infection, thus leading to a more variable response.

We have observed an increase in the numbers of macrophages (17) including AAMs (Figure 1 and K. Else, unpublished results) in the large intestines of mice with *T. muris* infection concordant with the development of Th2-mediated immunity. However, unlike the experiments with *H. bakeri* and *N. brasiliensis* infection, we observed no effects of arginase deficiency on *T. muris* expulsion with worm burdens in arginase-deficient mice equivalent to WT at day 35 post-infection. We used the mouse model (A*rg1*^*flox/flox*^*;Tie2-cre)* that ablates Arg1 preferentially from macrophages (9) as well as the arginase inhibitor, nor-NOHA (10), to assess the role of AAMs in *T. muris* expulsion. One explanation for the differences between our work with *T. muris* and published work using other gastrointestinal helminths (6) may be that clodronate deletes all macrophages irrespective of phenotype and arginase antagonists are not selective to the arginase-1 isoform and thus may be affecting additional pathways. Additionally, there may be regional differences in the actions of arginases within the intestinal tract. Furthermore, important differences exist between *N. brasiliensis*, *H. bakeri* and *T. muris* not least of which are the life cycles and niche in which the parasites reside.

A variety of Th2-regulated effector mechanisms are involved in the expulsion of *T. muris* (5) including increased muscle hypercontractility (5) and epithelial cell turn over (18). In *N. brasiliensis* infection, it was demonstrated that AAM had a major effector role in promoting smooth muscle hypercontractility and thus facilitating parasite expulsion from the small intestine (6). In contrast, we found that expulsion of *T. muris* was unaffected by depletion of AAMØ, suggesting that arginase-1 is not essential for resistance to *T. muris* and other mechanisms employed by the host immune system are sufficient for parasite expulsion. Interestingly, in *H. bakeri* infection, although depletion of AAMs was associated with impaired resistance to infection, it was also associated with reduced fecundity of the surviving parasites suggesting that, like *T. muris*, other AAM independent effector mechanisms exist. Similarly, the role of AAMs in *N. brasiliensis* infection is still unclear. Thus, mice with a macrophage-specific deficiency in IL-4Rα chain expression, which ablates the generation of AAM, were able to eliminate a *N. brasiliensis* infection as efficiently as wild-type mice (19). In contrast, clodronate-mediated depletion of macrophages and arginase inhibition have been shown to impair protective immunity to *N. brasiliensis* (6).

In addition to aiding host survival, Arg1 has been shown to down-regulate the inflammatory responses observed in response to infection. Arg1-expressing macrophages have been shown to act as suppressors of T cell-driven inflammation in *Schistosoma mansoni* infection (9,20). Pesce *et al.* used the transgenic mouse, A*rg1*^*flox/flox*^*tie2-cre,* to investigate the role of AAM in *S. mansoni* infection. Their work demonstrated a marked inhibition of T-cell proliferation that was restored by the addition of exogenous arginase; thus, they hypothesized that AAM locally depletes arginine within the granuloma environment and thereby inhibits Th2-mediated immune responses (9). However, in *T. muris* infection, we found no differences in the levels of Th2 cytokines such as IL-13, T-cell activity, antibody responses or number of inflammatory cells using either A*rg1*^*flox/flox*^*tie2-cre* mice or mice treated with the arginase inhibitor nor-NOHA. Unlike *T. muris* or *B. malayi*, the immune responses to *S. mansoni* infection are directed against the ova with the subsequent formation of granulomas in the liver and gut. Therefore, the different findings in these various parasitic infections may highlight the differences between the various parasite models studied, in terms of the model pathogen, life cycle and niche.

In addition to having a role in host resistance to infection and inhibiting Th2-mediated proliferation, AAMs have been hypothesized to be involved in suppressing Th1-driven infection-induced pathology in infection (12,20,21). High expression of Arg1 is associated with the formation of chronic nonhealing lesions in leishmaniasis and is thought to cause a local depletion of l-arginine, thus inhibiting local Th1 activity (21). Similarly, in *Toxoplasma gondii* infection, the induction of Arg1 favours parasite survival by inhibiting NO production and therefore parasite killing (12). These are examples in which the pathogens exploit the host immune response to favour their own survival. In acute schistosomiasis infection that is characterized by intestinal inflammation, there is an up-regulation of gene expression associated with AAMs along with genes involved in tissue remodelling (4,22). Mice deficient in macrophage Arg1 were used to show that Arg1 promotes production of TGFβ, suppresses the proinflammatory cytokines IL-12 and IL-23 p40 and thus favours the generation of regulatory T cells and inhibits local T-cell proliferation (20). *Trichuris muris* induces a mixed Th1 and Th2 immune response in C57BL/6 mouse strains with most mice having a dominant Th2 immune response and expelling the parasite. In a low-dose infection, however, a chronic infection develops characterized by a dominant Th1 immune response. We hypothesized that by abrogating Arg1 function in macrophages, we would see exacerbated infection-induced pathology in *T. muris* infection. To address this, we assessed mice in which there is a Th2-dominated resistance to *T. muris* infection as well as mice in which there is Th1-dominated chronic infection. However, analysis of cytokine responses, antibody titres and pathological indicators, including collagen staining, crypt cell hyperplasia, and goblet cell hyperplasia, failed to reveal any differences between mice with deficient Arg1 function in AAMs and wild-type controls post-infection. One possible explanation is that *T. muris* regulates host pathology directly through T cell-mediated mechanisms. For example, it has been recently shown that depletion of T-regulatory cells post-*T. muris* infection exacerbates helminth-induced pathology (23). While our data does not rule out macrophages as an important cell type in Th2-mediated immunity to *T. muris*, it does suggest that AAM-associated Arg1 is dispensable as an effector mechanism in both *T. muris* expulsion and *T. muris*-mediated pathology. This study shows that although immunity to different gastrointestinal helminths shares common features, including a CD4^+^ T-cell dependency, important differences exist in how host resistance is mediated and pathology modulated.
